# COVID-19 as a risk factor for long-term mortality in patients managed by the emergency medical system: A prospective, multicenter, ambulance-based cohort study

**DOI:** 10.3389/fpubh.2022.1076627

**Published:** 2023-01-10

**Authors:** José L. Martín-Conty, Begoña Polonio-López, Ancor Sanz-García, Carlos del Pozo Vegas, Laura Mordillo-Mateos, Juan José Bernal-Jiménez, Rosa Conty-Serrano, Miguel A. Castro Villamor, Raúl López-Izquierdo, Francisco Martín-Rodríguez

**Affiliations:** ^1^Faculty of Health Sciences, Universidad de Castilla-la Mancha, Talavera de la Reina, Spain; ^2^Technological Innovation Applied to Health Research Group (ITAS), Faculty of Health Sciences, University of Castilla-La Mancha, Talavera de la Reina, Spain; ^3^Prehospital Early Warning Scoring-System Investigation Group, Valladolid, Spain; ^4^Faculty of Medicine, Universidad de Valladolid, Valladolid, Spain; ^5^Emergency Department, Hospital Clínico Universitario, Valladolid, Spain; ^6^Faculty of Nursing, University of Castilla-La Mancha, Toledo, Spain; ^7^Emergency Department, Hospital Universitario Rio Hortega, Valladolid, Spain; ^8^Advanced Life Support, Emergency Medical Services (SACYL), Valladolid, Spain

**Keywords:** clinical decision rules, COVID-19, emergency medical services, long-term mortality, pre-hospital care

## Abstract

**Introduction:**

COVID-19 has initially been studied in terms of an acute-phase disease, although recently more attention has been given to the long-term consequences. In this study, we examined COVID-19 as an independent risk factor for long-term mortality in patients with acute illness treated by EMS (emergency medical services) who have previously had the disease against those who have not had the disease.

**Methods:**

A prospective, multicenter, ambulance-based, ongoing study was performed with adult patients with acute disease managed by EMS and transferred with high priority to the emergency department (ED) as study subjects. The study involved six advanced life support units, 38 basic life support units, and five emergency departments from Spain. Sociodemographic inputs, baseline vital signs, pre-hospital blood tests, and comorbidities, including COVID-19, were collected. The main outcome was long-term mortality, which was classified into 1-year all-cause mortality and 1-year in- and out-of-hospital mortality. To compare both the patients with COVID-19 vs. patients without COVID-19 and to compare survival vs non-survival, two main statistical analyses were performed, namely, a longitudinal analysis (Cox regression) and a logistic regression analysis.

**Results:**

Between 12 March 2020 and 30 September 2021, a total of 3,107 patients were included in the study, with 2,594 patients without COVID-19 and 513 patients previously suffering from COVID-19. The mortality rate was higher in patients with COVID-19 than in patients without COVID-19 (31.8 vs. 17.9%). A logistic regression showed that patients previously diagnosed with COVID-19 presented higher rates of nursing home residency, a higher number of breaths per minute, and suffering from connective disease, dementia, and congestive heart failure. The longitudinal analysis showed that COVID-19 was a risk factor for mortality [hazard ratio 1.33 (1.10–1.61); *p* < 0.001].

**Conclusion:**

The COVID-19 group presented an almost double mortality rate compared with the non-COVID-19 group. The final model adjusted for confusion factors suggested that COVID-19 was a risk factor for long-term mortality.

## Introduction

The current coronavirus disease 2019 (COVID-19) pandemic has been described as a novel severe acute respiratory syndrome coronavirus 2 (SARS-CoV-2), a disease condition at the beginning characterized by a massive number of cases, leading to unplanned intensive care unit (ICU) admissions, pneumonia with the multiorgan disease, and related mortality (particularly before mass vaccination programs) (1).

At the peak of the pandemic, a drop in notifications to emergency call centers for life-threatening diseases was observed, with a significant decrease in incidents attended by the emergency medical services (EMS) and the emergency department (ED) (2). A marked decrease in cases of acute myocardial infarction, stroke, or traffic accidents has also been reported (3–5), prioritizing COVID-19 (2). EMS were called upon to respond to biohazard medical emergencies, monopolizing patients with COVID-19 and virtually all ambulance transfers. Pre-hospital care was initially provided under unfavorable circumstances, e.g., the use of personal protective equipment, excessive evacuation delays, and, above all, a general unawareness concerning the transmission of the virus (6, 7).

With rapid tests, vaccinations, and effective therapies, the current pandemic has been kept under control, and health systems have managed to deal with COVID-19. We hypothesize that COVID-19 has likely been one of the factors, but not the unique one, of the exacerbation of chronic pathologies and of the observed over-mortality compared to the historical time series. This excess of mortality may result from the lack of appropriate and timely attention to life-threatening diseases, excess mortality due to COVID-19, or a combination of both circumstances (8).

Over the course of the outbreak, health systems have changed from assisting patients with COVID-19 and focusing all efforts on controlling the virus to assisting patients with diseases associated with COVID-19. In other words, COVID-19 has changed from being the primary disease to being treated for a patient in need of urgent care to being part of the full set of pathologies that may negatively affect the prognosis of the patient as a whole (9).

The objective of the present study was to compare long-term mortality (1-year mortality by all-cause and in- and out-of-hospital) in cases managed by EMS and subsequently transferred with high priority to ED in the following two contrasting prospective cohorts: cases with the acute disease without past COVID-19 vs. cases with the acute disease after COVID-19.

## Methods

### Study design and settings

The present prospective, multicenter, ambulance-based, ongoing study included adult patients with acute disease managed by EMS and transferred with high priority to the ED, collected from two back-to-back prospective studies carried out under the same operative guideline from 12 March 2020 to 30 September 2021.

The study was carried out in four Spanish provinces, i.e., Burgos, Salamanca, Segovia, and Valladolid, covering 24/7 urban, suburban, and rural areas with a reference population of 1,166,746 inhabitants, involving the coordination center 1-1-2, six advanced life support units (ALSU), 38 basic life support units (BLSU), and five EDs, resources managed by the regional public health system (SACYL).

The study protocol was registered in the WHO International Clinical Trials Registry Platform (ISRCTN48326533 and ISRCTN49321933), was approved by the institutional review board of public health (reference: PI-049-19/PI-GR-19-1258), and followed the STrengthening the Reporting of OBservational studies in Epidemiology (STROBE) (Supplementary material) (10). Written informed consent was obtained from all the study participants at the EMS attendance. Patients without informed consent were excluded.

### Population

In this study, two prospective cohorts were established. Cohort #01 included acute disease cases with no prior history of COVID-19. Cohort #2 was composed of acute disease cases who had been previously infected by COVID-19.

Adult patients (≥18 years) with acute disease, assisted consecutively by an ALSU and evacuated to ED by ALSU or BLSU, with a 1-year follow-up period were included. Those patients who present the following exclusion criteria were not considered in the study: patients with active COVID-19 cases (this exclusion criterion was selected to avoid the effect of acute infection and to focus on the long-term effects of the previous infection), patients aged <18 years, patients who had cardiorespiratory arrest (on the scene or *en route*), patients who were terminally ill (documented condition), pregnant women, cases discharged *in situ*, and patients with <1-year follow-up. The sample size was based on an opportunity sample method, i.e., selecting all the patients who met the criteria during the study time.

### Outcome

The main outcome was long-term mortality, which was classified into 1-year all-cause mortality and 1-year in- and out-of-hospital mortality after the ambulance transfer. The 1-year follow-up period was in line with comparable studies (11, 12). The principal outcome was blinded to the clinical researchers responsible for collecting the data. As the electronic health record is linked to the community mortality registry, all deaths, even those that occurred out-of-hospital, were included in the study. The outcome was retrieved at the end of the study follow-up.

### Measures

Sociodemographic inputs (sex, age, urban/rural area, nursing home residence, and evacuation way to the hospital) were collected by an ALSU emergency medical technician. Baseline vital signs (respiratory rate—number of breaths per minute, oxygen saturation, pulse oximetry saturation/fraction of inspired oxygen ratio, blood pressure, heart rate, temperature, and Glasgow Coma Scale) and pre-hospital blood tests (glucose, lactate, and creatinine) were picked up and recorded by the ALSU emergency registered nurse during the first contact with the patient, either at the scene or *en route*. Oxygen saturation, blood pressure (systolic, diastolic, and mean), and heart rate was obtained using LifePAK^®^ 15 monitor-defibrillator (Physio-Control, Inc., Redmond, USA), and temperature using ThermoScan^®^ PRO 6000 thermometer (Welch Allyn, Inc., Skaneateles Falls, USA). The analytical blood test was carried out using point-of-care testing epoc^®^ Blood Analysis System (Siemens Healthcare GmbH, Erlangen, Germany). Finally, the ALSU physician compiled the pre-hospital advanced life support special follow-up procedures, namely, non-invasive respiratory support, invasive respiratory support, and/or use of vasoactive medications (norepinephrine), as well as the pre-hospital presumptive diagnosis, updated based on the 11th revision of the International Classification of Diseases.

To correctly match EMS and the electronic medical record of a hospital patient, we required the exact linkage of at least 5 identifiers, including date, admission time in ED, age, sex, ambulance code, name and surname, and/or healthcare card number. Upon data de-screening, an exact linkage failed with at least five identifiers out of 39 cases, which were excluded from the final analysis.

To assess in-hospital variables, an associate investigator assigned to each hospital (with pre-hospital care records blinded) captured the following at the end of follow-up: SARS-CoV-2 positives (polymerase chain reaction and/or rapid antigen test), 17 categories of comorbidities required to calculate the age-adjusted Charlson comorbidity index (aCCI) (myocardial infarction, congestive heart failure, peripheral vascular disease, stroke or transient ischemic attack, dementia, chronic obstructive pulmonary disease, connective tissue disease, peptic ulcer disease, mild liver disease, uncomplicated diabetes mellitus, hemiplegia, moderate to severe chronic kidney disease, diabetes mellitus with end-organ damage, localized solid tumor, leukemia, lymphoma, moderate to severe liver disease, metastatic solid tumor, and AIDS), hospitalization, ICU admission, and 1-year mortality (all-cause and in- and out-of-hospital). Finally, a data manager calculated the modified sequential organ failure assessment (mSOFA) (13) and aCCI scores (14).

### Statistical analysis

Percentages were used to represent categorical variables, and the mean and standard deviation was used as continuous variables. All the comparisons followed the same procedure: first, a univariate comparison, followed by a multivariate regression using those variables with a *p*-value of <0.001. In particular, two main factors were used to compare groups: patients who had COVID-19 or patients without COVID-19 and mortality. This comparison was performed by considering the whole cohort, selecting only those patients who died or selecting those patients who previously suffered from COVID-19.

A comparison between patients with COVID-19 and patients without COVID-19 for the whole cohort and for those who died within the follow-up time was performed using the Mann-Whitney U test, *T*-test, or chi-squared test, when appropriate, followed by logistic regression with a forward and backward stepwise variable selection. The comparison for mortality was performed by the log rank followed by Cox regression. Furthermore, the survival according to patients with COVID-19 or patients without COVID-19 was obtained using the Kaplan-Meier method (KM).

Data were analyzed using our own codes and basic functions in R, version 4.2.1 (http://www.R-project.org; the R Foundation for Statistical Computing, Vienna, Austria).

## Results

**Figure 1:**
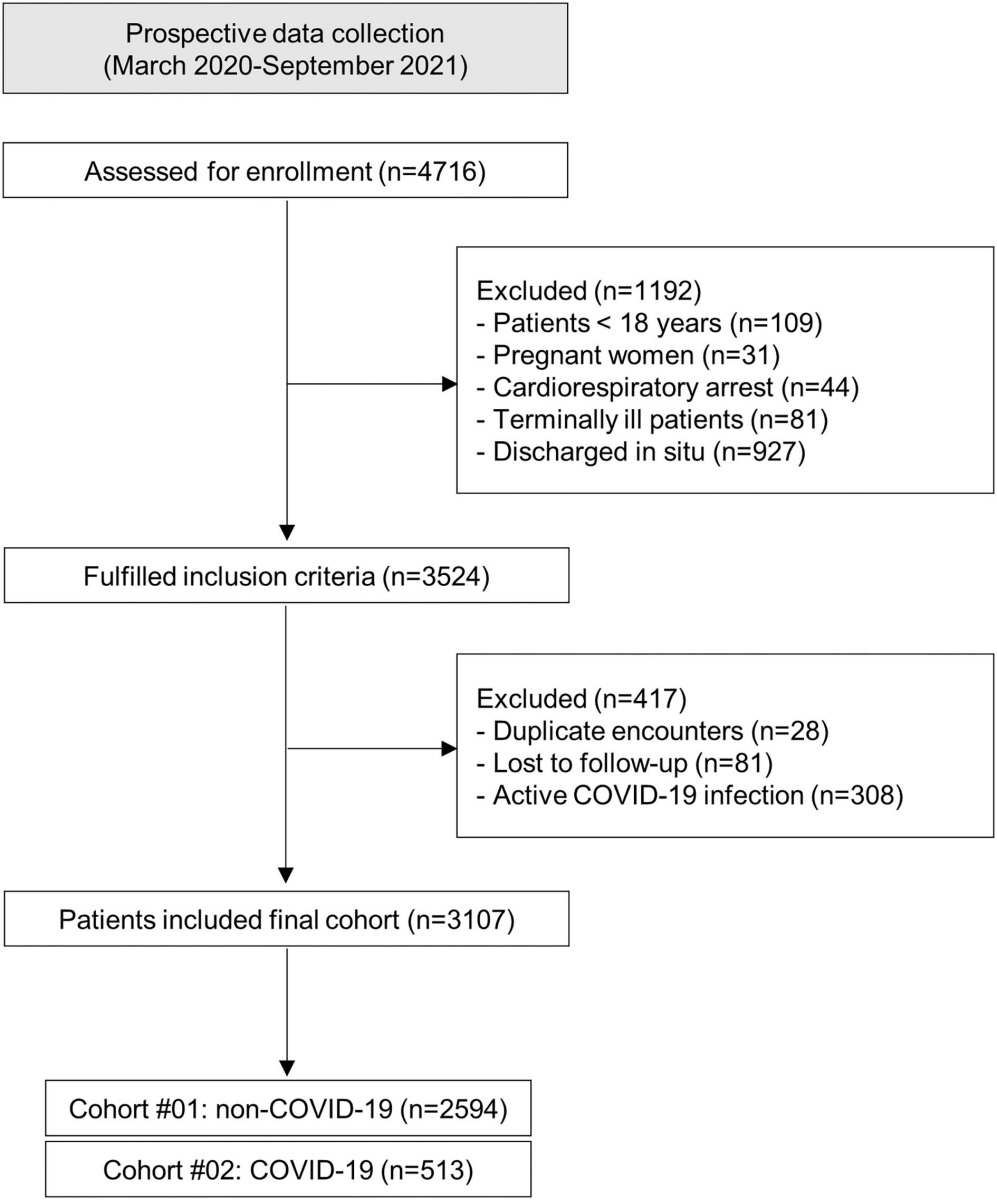
Study flowchart. COVID-19, coronavirus disease 2019.

A total of 3,107 patients with acute disease managed by pre-hospital care and referred to the ED were included in the final evaluation: 2,594 in cohort #01 (non-COVID-19) and 513 in cohort #02 (COVID-19). We excluded 308 confirmed active COVID-19 cases in ED (Figure 1).

The median age was 67 years (IQR (interquartile range): 50–81 years), with 41.8% women (1,299 cases). Demographic characterization by COVID-19 cohort included older adults evacuated by ALSU mainly from urban areas to ED and derived to a large extent from nursing homes, with a significant number of comorbidities (especially congestive heart failure, myocardial infarction, dementia, connective disease, and severe chronic kidney disease). The non-COVID-19 cohort exhibited a similar median age, with more middle-aged cases, significantly reduced comorbidities, and a lower nursing home origin. Clinically, both groups reported the same qSOFA, and a similar percentage of pre-hospital advanced life support special procedures, with comparable hospitalization and ICU admission rates (Tables 1, 2).

**Table 1:** Demographic characteristics.

		**Associated comorbidity**			
**Variable**	**Total**	**COVID-19**	**Non-COVID-19**	**Standardized difference^b^**	***p*-value^c^**
No. (%) with data^a^	3,107	513 (16.5)	2,594 (83.5)	N.A.	N.A.
Age, year	67 (50–81)	74 (56–83)	66 (50–80)	0.264	<0.001
Age groups, year^d^				0.219	<0.001
18–49	726 (23.4)	92 (17.9)	634 (24.4)		
50–74	1,203 (38.7)	183 (35.7)	1,020 (39.3)		
>75	1,178 (37.9)	238 (46.4)	940 (36.2)		
Sex, women	1,299 (41.8)	237 (46.2)	1,062 (40.9)	0.106	0.027
ALS	1,992 (64.1)	302 (58.9)	1,690 (65.2)	0.13	0.007
Urban area resident	2,233 (71.9)	396 (77.2)	1,837 (70.8)	0.146	0.003
Nursing homes resident	305 (9.8)	99 (19.3)	206 (7.9)	0.335	<0.001
**Basal vital signs**					
RR, number of breaths/min	17 (14–23)	19 (15–26)	17 (14–22)	0.29	<0.001
SpO_2_, %	96 (94–98)	96 (92–98)	97 (94–98)	0.254	<0.001
FiO_2_, %	0.21 (0.21–0.21)	0.21 (0.21–0.21)	0.21 (0.21–0.21)	0.019	0.689
SaFi	457 (443–467)	452 (429–467)	457 (443–467)	0.179	<0.001
SBP, mmHg	133 (113–151)	131 (107–151)	133 (114–151)	0.104	0.035
DBP, mmHg	78 (65–90)	77 (61–88)	78 (65–90)	0.14	0.004
MBP, mmHg	96 (83–109)	94 (79–108)	97 (83–110)	0.132	0.007
HR, number of beats/min	84 (70–103)	87 (70–105)	83 (70–102)	0.1	0.043
Temperature, °C	36.1 (35.9–36.6)	36.1 (35.8–36.7)	36.1 (35.9–36.6)	0.028	0.589
GCS, points	15 (15–15)	15 (14–15)	15 (15–15)	0.101	0.044
Glucose, mg/dL	130 (106–164)	135 (109–180)	128 (105–160)	0.111	0.022
Creatinine, mg/dL	0.92 (0.76–1.22)	0.98 (0.77–1.41)	0.91 (0.76–1.18)	0.149	0.002
Lactate, mmol/L	2.08 (1.23–3.21)	2.33 (1.36–3.32)	2.07 (1.21–3.18)	0.095	0.041
**Outcomes**					
mSOFA, points	1 (0–3)	1 (0–4)	1 (0–3)	0.159	0.002
NIRS	92 (3)	27 (5.3)	65 (2.5)	0.143	0.001
IRS	191 (6.1)	33 (6.4)	158 (6.1)	0.014	0.768
Noradrenaline use	82 (2.6)	22 (4.3)	60 (2.3)	0.111	0.011
Hospital-inpatient	1,701 (54.7)	308 (60)	1,393 (53.7)	0.128	0.008
Hospitalization-day	2 (0–8)	2 (0–8)	2 (0–7)	0.07	0.158
ICU-admission	329 (10.6)	57 (11.1)	272 (10.5)	0.02	0.674
1-year mortality	629 (20.3)	163 (31.8)	466 (17.9)	0.323	<0.001

NA, not applicable; ALSn, advanced life support requirement; RR, respiratory rate: SPO_2_, oxygen saturation; FiO_2_, fraction of inspired oxygen; SaFi, pulse oximetry saturation/fraction of inspired oxygen ratio; SBP, systolic blood pressure; DBP, diastolic blood pressure; MBP, mean blood pressure {MBP = [(2 × DBP) + SBP] / 3}; HR, heart rate; GCS, Glasgow Coma Scale; mSOFA, modified sequential organ failure assessment; NIRS, non-invasive respiratory support; IRS, invasive respiratory support; hospital-inpatient (admission to hospital); hospitalization-day (days of hospitalization); ICU, intensive care unit; COVID-19, coronavirus disease.

a Values expressed as total number (fraction) and medians [25 percentile-75 percentile], as appropriate.

b The Cohen's d-test was used to estimate the effect size.

c The Mann–Whitney U test, T-test, or chi-squared test were used as appropriate.

d The age group selection was based on both epidemiological and statistical criteria, i.e., our distribution of patients across groups.

**Table 2:** Comorbidities of baseline patients and diagnosis group.

		**Associated comorbidity**			
**Variable**	**Total**	**COVID-19**	**Non-COVID-19**	**Standardized difference^b^**	***p*-value^c^**
No. (%) with data^a^	3,107	513 (16.5)	2,594 (83.5)	N.A.	N.A.
Diagnosis group				0.046	0.349
Cardiovascular	1,149 (37)	187 (36.5)	962 (37.1)		
Neurology	562 (18.1)	80 (15.6)	482 (18.6)		
Respiratory	211 (6.8)	56 (10.9)	155 (6)		
Digestive	131 (4.2)	30 (5.8)	101 (3.9)		
Infection	183 (5.9)	48 (9.4)	135 (5.2)		
Trauma and injury	541 (17.4)	71 (13.8)	470 (18.1)		
Poisoning	250 (8)	26 (5.1)	224 (8.6)		
Others^d^	80 (2.6)	15 (2.9)	65 (2.5)		
aCCI (points)	2 (0–5)	3 (1–6)	1 (0–4)	0.252	<0.001
AIDS	38 (1.2)	12 (2.3)	26 (1)	0.105	0.012
Solid tumor metastatic	118 (3.8)	25 (4.9)	93 (3.6)	0.064	0.063
Liver disease severe	112 (3.6)	29 (5.7)	83 (3.2)	0.119	0.06
Lymphoma	35 (1.1)	4 (0.8)	31 (1.2)	0.042	0.416
Leukemia	31 (1)	7 (1.4)	24 (0.9)	0.041	0.360
Solid tumor localized	498 (16)	97 (18.9)	401 (15.5)	0.091	0.052
DM end organ damage	316 (10.2)	58 (11.3)	258 (9.9)	0.044	0.352
Severe CKD	304 (9.8)	70 (13.6)	234 (9)	0.146	0.001
Hemiplegia	129 (4.2)	34 (6.6)	95 (3.7)	0.134	0.002
DM uncomplicated	376 (12.1)	77 (15)	299 (11.5)	0.103	0.027
Liver disease mild	105 (3.4)	25 (4.9)	80 (3.1)	0.092	0.040
Peptic ulcer disease	276 (8.9)	61 (11.9)	215 (8.3)	0.12	0.009
Connective disease	187 (6)	51 (9.9)	136 (5.2)	0.178	<0.001
COPD	643 (20.7)	123 (24)	520 (20)	0.095	0.045
Dementia	287 (9.2)	84 (16.4)	203 (7.8)	0.264	<0.001
Cerebrovascular disease	287 (9.2)	56 (10.9)	231 (8.9)	0.067	0.151
Peripheral vascular disease	319 (10.3)	60 (11.7)	259 (10)	0.055	0.243
Congestive heart failure	441 (14.2)	130 (25.3)	311 (12)	0.348	<0.001
Myocardial infarction	582 (18.7)	117 (22.8)	465 (17.9)	0.121	0.010

NA, not applicable; aCCI, age-adjusted Charlson comorbidity index; AIDS, acquired immunodeficiency syndrome; DM, diabetes mellitus; CKD, chronic kidney disease; COPD, chronic obstructive pulmonary disease; ICU, intensive care unit; COVID-19, coronavirus disease.

a Values expressed as total number (fraction) and medians [25 percentile-75 percentile], as appropriate.

b The Cohen's d-test was used to estimate the effect size.

c The Mann–Whitney U test, T-test, or chi-squared test were used as appropriate.

d Other pathology: endocrine, genitourinary, diseases of the blood, and the immune system.

**Figure 2:**
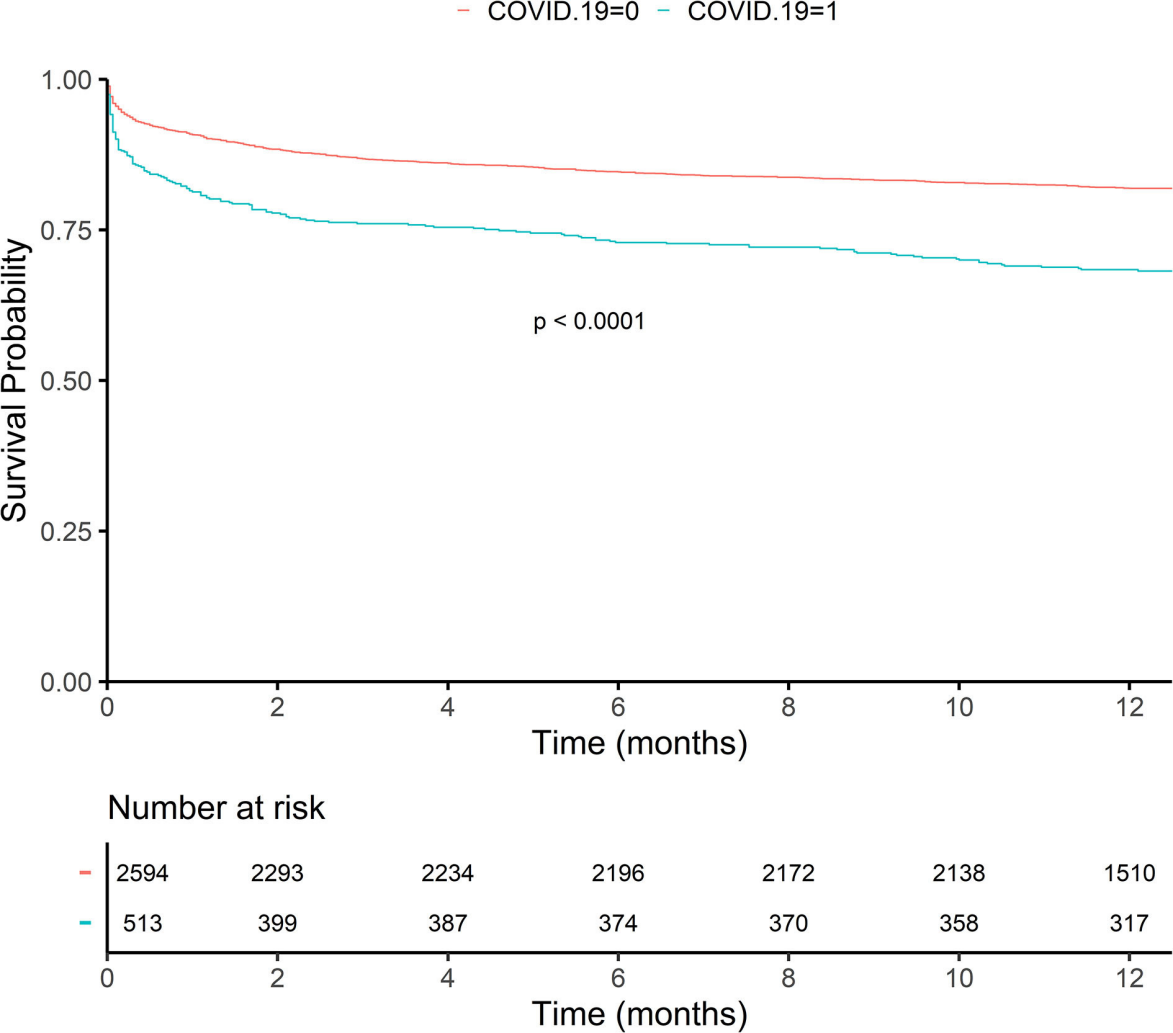
The Kaplan–Meier curve showing the survival probability for patients with and without COVID-19. The red line represents patients without COVID-19. The blue line represents patients with COVID-19.

The overall 1-year mortality was 20.3% (629 cases). Comparing both cohorts, the mortality rate in the COVID-19 group was 13.9 points higher than the one in the non-COVID-19 group (31.8 vs. 17.9%). Cumulative mortality by time points, respectively, 1, 2, 7, 30, 90, 180, and 365 days, in the COVID-19 cohort increased consistently over all time points, exhibiting about double the cumulative mortality vs. the non-COVID-19 cohort for all the time points (Table 3). This result was corroborated by the KM curve (Figure 2); as can be observed, both groups remained parallel throughout the follow-up.

**Table 3:** Outcomes of long-term mortality patients.

	**1-year mortality**			
**Variable**	**COVID-19**	**Non-COVID-19**	**Standardized difference^b^**	***p*-value^c^**
Cumulative mortality^a^			N.A.	N.A.
1-day	30 (5.8)	75 (2.8)	0.061	<0.001
2-day	45 (8.7)	104 (4.1)	0.122	<0.001
7-day	65 (12.6)	159 (6.1)	0.124	<0.001
30-day	96 (18.7)	239 (9.2)	0.153	<0.001
90-day	123 (23.9)	343 (13.2)	0.042	<0.001
180-day	139 (27.1)	399 (15.3)	0.01	<0.001
In-hospital	88 (54)	234 (50.2)	0.075	0.407
Out-hospital	75 (46)	232 (49.8)	0.075	0.407
Age, year	81 (71–88)	79 (66–86)	0.203	0.023
Age groups, year^e^			0.132	0.146
18–49	11 (6.7)	28 (6)		
50–74	38 (23.3)	153 (33.8)		
>75	114 (69.9)	285 (61.2)		
Sex, female	72 (44.2)	171 (36.7)	0.152	0.092
ALS	111 (68.1)	325 (69.7)	0.035	0.696
Urban area resident	124 (76.1)	349 (74.9)	0.027	0.764
Nursing homes resident	59 (36.2)	84 (18)	0.417	<0.001
Pre-hospital outcomes				
mSOFA, points	4 (2–7)	4 (2–6)	0.065	0.479
NIRS	18 (11)	40 (8.6)	0.083	0.351
IRS	25 (15.3)	90 (19.3)	0.105	0.259
Noradrenaline use	20 (12.3)	41 (8.8)	0.113	0.198
Diagnosis group			0.035	0.697
Cardiovascular	57 (35)	132 (28.3)		
Neurology	20 (12.3)	99 (21.2)		
Respiratory	27 (16.6)	67 (14.4)		
Digestive	9 (5.5)	28 (6)		
Infection	24 (14.7)	53 (11.4)		
Trauma and injury	18 (11)	59 (12.7)		
Poisoning	2 (1.2)	11 (2.4)		
Others^d^	6 (3.7)	17 (3.6)		
aCCI (points)	5 (3–9)	4 (2–8)	0.162	0.013
Hospital-inpatient	135 (82.8)	394 (84.5)	0.047	0.604
Hospitalization-day	4 (1–9)	5 (1–12)	0.133	0.172
ICU-admission	27 (16.6)	109 (23.4)	0.171	0.069

NA, not applicable; ALS, advanced life support requirement; mSOFA, modified sequential organ failure assessment; NIRS, non-invasive respiratory support; IRS, invasive respiratory support; aCCI, age-adjusted Charlson comorbidity index; Hospital-inpatient, admission to hospital; Hospitalization-day, days of hospitalization; ICU, intensive care unit; COVID-19, coronavirus disease.

a Values expressed as total number (fraction) and medians [25 percentile-75 percentile], as appropriate.

b The Cohen's d-test was used to estimate the effect size.

c The Mann–Whitney U test, T-test, or chi-squared test were used as appropriate.

d Other pathology: endocrine, genitourinary, diseases of the blood, and the immune system.

e The age group selection was based on both epidemiological and statistical criteria, i.e., our distribution of patients across groups.

When considering the whole cohort (Table 4A) or only those with 1-year mortality (Table 4B), the logistic multivariate analysis of COVID-19 vs. non-COVID-19 showed that the main characteristics of patients with COVID-19 were being in a nursing home and suffering from dementia or congestive heart failure. Additionally, when considering the whole cohort (Table 4A), patients with COVID-19 suffered from connective disease, presented a higher number of breaths per minute, and had higher 1-year mortality.

**Table 4:** Multivariate logistic regression for patients with COVID-19 vs. patients without COVID-19.

**Variable**	**Odds ratio (95%CI)**	***p*-value**
**(A) Whole cohort**		
Nursing homes resident	1.74 [1.35–2.24]	<0.001
RR, number of breaths/min	1.02 [1.01–1.03]	<0.001
Connective disease	1.63 [1.21–2.18]	<0.001
Dementia	1.44 [1.10–1.87]	<0.001
Congestive heart failure	1.80 [1.46–2.22]	<0.001
1-year mortality	1.45 [1.19–1.76]	<0.001
**(B) Selecting those patients with 1-year mortality**		
Nursing homes resident	2.04 [1.41–2.95]	0.001
Dementia	1.55 [1.05–2.28]	0.06
Congestive heart failure	1.91 [1.38–2.64]	<0.001

RR, respiratory rate; 95% CI, 95% confidence interval.

Similar to the comparison between patients with COVID-19 and patients without COVID-19, the longitudinal analysis of mortality for the whole cohort (Table 5) showed that factors associated with mortality included (results from Cox regression) age, respiratory support both invasive and non-invasive, noradrenaline administration, hospital admission, and hospital stay duration. The diagnosis groups that stood out as risk factors were respiratory, digestive, infection, and trauma and injury. Pathologies associated with mortality were a metastatic solid tumor, leukemia, and congestive heart failure. Those patients with COVID-19 presented a higher risk of mortality, a variable that remains statistically significant despite the high number of confounding factors. Finally, the mSOFA score was higher in those patients with a higher risk of mortality, suggesting its reliability in predicting clinical worsening even at long-term follow-ups. Further details of the results from this analysis can be found in Supplementary Table S1.

**Table 5:** Factors associated with mortality (univariate and multivariate by Cox regression) for the whole cohort.

	**Univariate (log-rank)**		**Multivariate (Cox regression)**	
**Variable**	**Hazard ratio (95%CI)**	***p*-value**	**Hazard ratio (95%CI)**	***p*-value**
Age, year	1.04 [1.04;1.05]	<0.001	1.03 [1.01–1.05]	<0.001
mSOFA, points	1.40 [1.37;1.43]	<0.001	1.28 [1.18–1.38]	<0.001
NIRS	4.85 [3.70;6.35]	<0.001	2.06 [1.46–2.91]	<0.001
IRS	5.79 [4.73;7.09]	<0.001	2.93 [2.03–4.23]	<0.001
Noradrenaline use	9.46 [7.25;12.3]	<0.001	1.70 [1.21–2.39]	0.002
Respiratory (diagnosis group)	3.13 [2.45;4.01]	<0.001	2.06 [1.52–2.78]	<0.001
Digestive (diagnosis group)	1.79 [1.26;2.55]	0.001	1.87 [1.28–2.73]	0.001
Infection (diagnosis group)	3.04 [2.33;3.96]	<0.001	1.96 [1.43–2.68]	<0.001
Trauma and injury (diagnosis group)	0.87 [0.67;1.14]	0.313	1.65 [1.22–2.22]	0.001
Solid tumor metastatic	4.41 [3.46;5.63]	<0.001	4.28 [3.20–5.73]	<0.001
Leukemia	3.34 [2.03;5.49]	<0.001	4.21 [2.49–7.11]	<0.001
Congestive heart failure	2.85 [2.40;3.39]	<0.001	1.52 [1.25–1.85]	<0.001
Hospital-inpatient	5.16 [4.17;6.39]	<0.001	2.43 [1.90–3.10]	<0.001
Hospitalization-day	1.01 [1.01;1.02]	<0.001	0.97 [0.96–0.98]	<0.001
COVID-19	1.93 [1.62;2.31]	<0.001	1.33 [1.10–1.61]	<0.001

Only statistically significant variables or categories of variables are shown.

95% CI, 95% confidence interval; mSOFA, modified sequential organ failure assessment; NIRS, non-invasive respiratory support; IRS, invasive respiratory support; Hospital-inpatient, admission to hospital; Hospitalization-day, days of hospitalization; COVID-19, coronavirus disease.

To determine the factors critical for mortality for patients with COVID-19, the same procedure applied in the previous analysis was used for the cohort of patients with COVID-19 (Table 6); further details of these results can be found in Supplementary Table S2. Again, age, mSOFA, respiratory disease, metastatic solid tumor, leukemia, and congestive heart failure were risk factors for mortality. This more detailed analysis showed that hemiplegia, high aCCI, diastolic blood pressure, and FiO_2_ were critical factors for mortality within the COVID-19 group.

**Table 6:** Factors associated with mortality (univariate and multivariate by Cox regression) for those patients with COVID-19.

	**Univariate (log-rank)**		**Multivariate (Cox regression)**	
**Variable**	**Hazard ratio (95%CI)**	***p*-value**	**Hazard ratio (95%CI)**	***p*-value**
Age, year	1.04 [1.03;1.06]	<0.001	1.06 [1.02–1.11]	0.001
FiO_2_, %	10.5 [3.52;31.5]	<0.001	0.01 [0.00–0.50]	0.020
DBP, mmHg	0.98 [0.98;0.99]	<0.001	1.01 [1.00–1.02]	0.025
Outcomes				
mSOFA, points	1.39 [1.33;1.45]	<0.001	1.40 [1.17–1.68]	<0.001
Noradrenaline use	10.5 [6.50;17.0]	<0.001	2.88 [1.37–6.06]	0.005
Respiratory (diagnosis group)	1.77 [1.12;2.80]	0.014	2.41 [1.30–4.45]	0.004
aCCI (points)				
0	Ref.	Ref.		
1	0.80 [0.30;2.16]	0.659	0.25 [0.07–0.84]	0.025
2	2.63 [1.24;5.56]	0.012	0.28 [0.08–0.98]	0.048
3	2.85 [1.45;5.59]	0.002	0.20 [0.04–0.88]	0.033
Solid tumor metastatic	2.90 [1.76;4.80]	<0.001	3.73 [1.95–7.12]	<0.001
Leukemia	7.59 [3.53;16.3]	<0.001	10.0 [3.86–26.0]	<0.001
Hemiplegia	3.00 [1.93;4.67]	<0.001	1.75 [1.00–3.04]	0.046
Congestive heart failure	2.45 [1.79;3.35]	<0.001	2.02 [1.33–3.05]	<0.001

Only statistically significant variables or categories of variables are shown.

95% CI, 95% confidence interval; Ref, reference category for hazard ratio calculation; FiO_2_, fraction of inspired oxygen; DBP, diastolic blood pressure; mSOFA, modified sequential organ failure assessment; aCCI, age-adjusted Charlson comorbidity index.

## Discussion

The massive caseload caused by SARS-CoV-2 has consequently led to an increase in mortality rates, associated both with the pandemic and with the suboptimal support provided to non-COVID-19 disease at the start of the outbreak.

Patients treated by pre-hospital care without COVID-19 (cases with an acute disease that did not present the previous COVID-19) showed a 1-year mortality rate close to 18%. According to our results, 1-year mortality for those from the COVID-19 group (cases formerly infected by COVID-19) was 13.9 points higher. A longitudinal analysis showed that presenting COVID-19 as an antecedent is a risk factor for long-term mortality.

Chronic preexisting health conditions are well-documented to play a key role in long-term survival; the greater the number of pathologies, the lower the likelihood of survival and the higher the likelihood of in-patient hospitalization, rehospitalization, and ICU admission rates (15, 16). The number of pathologies was observed as a key factor for short-, medium-, and long-term related mortality since the beginning of the pandemic (17, 18). Different studies examined long-term mortality in post-COVID-19 patients (19–21), but to the best of our knowledge, no research has analyzed the impact of COVID-19 as a previous condition among acute disease patients managed in pre-hospital care.

This over-mortality, according to our study, appears to have a multi-causal explanation. The cases included were multi-pathological patients, such as cardiovascular and neurologic diseases or trauma and injury. Pre-hospital care was homogeneous among both cohorts in terms of assessment using the mSOFA (13) (pulse saturation/inspired oxygen fraction ratio, mean arterial pressure, Glasgow Coma Scale, creatinine, and lactate), although some advanced life support techniques were preferred in COVID-19 cohort, e.g., non-invasive mechanical ventilation and noradrenaline use (22). The rates of hospital-inpatient, hospitalization-day, and ICU admission were statistically equivalent.

The above results reinforce the argument that over-mortality could be caused by a combination of variables. Chronological age is an unquestionable biological factor. In addition, chronological age plays a pivotal role in chronic diseases, so the older the age, the increased the comorbidities (23, 24). Despite age showing significant differences between groups, we believe that comorbidity burden was the most decisive factor since age was not statistically significant in the multivariate logistic regression. The COVID-19 cohort exhibited a median aCCI of 3 points vs. 1 point in the non-COVID-19 cohort. A detailed analysis highlighted an increase observed in cardiovascular pathology (congestive heart failure and myocardial infarction) and dementia in the COVID-19 cohort, with data in line with similar studies (25–27), since those conditions are associated with common exacerbations, hospital-inpatient, and ultimately poor long-term outcomes. Other pulmonary diseases, such as chronic obstructive pulmonary disease, were not related to a significant increase in 1-year mortality with similar results in both cohorts (28).

Nursing home affiliation was a critical factor directly involved in the mortality of the COVID-19 cohort. Remarkably, at the start of the outbreak, unacceptable mortality rates associated with nursing homes were observed. Admittedly, patients are multi-pathological, with multiple comorbidities, and generally of elderly age, but the over-mortality described in nursing homes should give us a wake-up call to reconsider this fact as a healthcare system (29, 30). Nursing home mortality was two times as high as in the COVID-19 cohort compared to patients managed by EMS due to acute disease without COVID-19; this irrefutable observation flags nursing home affiliation as a critical factor underlying poor long-term outcomes (31).

The above-mentioned results suggest that COVID-19 plays an important role in this long-term mortality, and three main reasons could be argued for the importance of COVID-19 in long-term mortality: First, in the selection of patients, all the patients were selected based on an opportunity sample method, i.e., selecting all the patients who accomplished criteria during the study time. The difference between the COVID-19 and non-COVID-19 groups regarding age or comorbidities was due to chance rather than a consequence of having suffered from COVID-19. When using the above-mentioned confusion factors in the Cox regression (Table 5), none of them (and the other confusion factors) exclude COVID-19 as a risk factor for mortality. In addition, when all statistically significant factors (including age and aCCI) were adjusted in a regression model to determine the final model that described the difference between the COVID-19 and non-COVID-19 groups (Table 4), age and aCCI were automatically (by the regression algorithm) excluded from the final model, and only a few comorbidities alone were included. Epidemiological studies have shown an excess of mortality in patients with COVID-19 compared to analogous historical series (32, 33). Even though mortality also increased in patients without COVID-19 in the early stages of the pandemic, this trend has gradually normalized to previous levels as healthcare returned to pre-pandemic attention levels and due to the improvement of COVID-19 handling (34). Therefore, as the pandemic evolved, one should expect a reduction in mortality, which was not the case according to our results.

Our study is not free of limitations. First, a pure convenience sample was collected consecutively. To control for potential bias, data input was gathered 24/7 non-stop throughout the study period in ambulance stations located in urban, suburban, and rural areas, patients transferred to ED of different hospitals, and hospitals with different clinical qualifications, attempting to be a true cross-section of the analyzed population. Second, the data extractors were not blinded. To avoid cross-contamination, the EMS staff was unaware of the scores being estimated and interpreted, and as a double fail-safe, the research associates from each hospital were unaware of the pre-hospital parameters as well. Only the data manager and the principal investigator could access the master database. Third, confirmed cases of COVID-19 were taken as patients with a positive polymerase chain reaction and/or rapid antigen test, but an underestimation is possible. Currently, some people skip screening or do not report self-test results. At the onset of the outbreak, the availability of test kits was limited, even though the incidence rates should be treated with caution. In this sense, antibody tests for the non-COVID-19 group were not available, so it cannot be completely ruled out that they did not have COVID-19. Fourth, the study was carried out across different provinces, all of which comprise the same health system. To validate the findings, multicenter studies in different regions involving several institutions should be carried out. Fifth, in the present study, we did not consider all the patients who could present long-term mortality; this is because patients could reach the emergency department by their means without requiring assistance from EMS. However, this study aimed to focus on patients who required pre-hospital emergency care. Sixth, since this study has been developed in the pre-hospital scenario, critical factors related to the long-term consequences of COVID-19 have not been considered due to the impossibility to achieve them, for instance: the date of infection (hampering determining the time between infection and the EMS attendance), the severity of COVID-19, the treatment the COVID-19 (whether it required intensive therapy or invasive mechanical ventilation), and the treatment after COVID-19 hospitalization. Seventh, the duration or diagnostic time of comorbidities was not available; however, despite being important information regarding the status of the patients, it is not included in the commonly used comorbidity-based scores.

## Conclusion

According to our results, the COVID-19 group presented a higher mortality rate than the non-COVID-19 group. The predictive model, when adjusted by confusion factors, showed COVID-19 as a relevant risk factor for mortality.

## Data availability statement

The raw data supporting the conclusions of this article will be made available by the authors, without undue reservation.

## Ethics statement

The studies involving human participants were reviewed and approved by the Institutional Research Review Board of each health area (reference: PI-049-19/PI-GR-19-1258). The patients/participants provided their written informed consent to participate in this study.

## Author contributions

FM-R conceptualized the project, managed and coordinated the project, assisted with the design of methodology, analyzed data, and prepared the initial and final drafts of the manuscript. AS-G takes responsibility for the data and their analysis. LM-M, JB-J, RC-S, BP-L, CP, MC, and JM-C assisted with the management and coordination of the project, assisted with the design of methodology, and helped review the manuscript. RL-I conceptualized the project and helped review and comment on the initial and final drafts of the manuscript. All authors performed a critical review and approved the final manuscript for interpretation of the data and important intellectual input.
