# Supplementary material for: COVID-19 as a risk factor for long-term mortality in patients managed by the emergency medical system: A prospective, multicenter, ambulance-based cohort study
Source: Front Public Health. 2023 Jan 10;10:1076627. doi: 10.3389/fpubh.2022.1076627 (PMC9871910; doi:10.3389/fpubh.2022.1076627)
Supplement: Supplementary file 1 [file Data_Sheet_1.docx]

**Supplementary Material**

Table S1. Factors associated to mortality (univariate and multivariate by Cox regression) for the whole cohort.

|  | Univariate (log-rank) | | Multivariate (Cox regression) | |
| --- | --- | --- | --- | --- |
| Variable | Hazard ratio (95%CI) | *p* value | Hazard ratio (95%CI) | *p* value |
| Age, year | 1.04 [1.04;1.05] | <0.001 | 1.03 [1.01-1.05] | <0.001 |
| Age groups, year^a^ |  |  | Not Included |  |
| 18-49 | Ref. | Ref. |  |  |
| 50-74 | 3.11 [2.21;4.39] | <0.001 |  |  |
| >75 | 7.44 [5.35;10.3] | <0.001 |  |  |
| Sex, Female | 0.86 [0.73;1.01] | 0.067 | Not Included |  |
| ALS | 0.75 [0.63;0.88] | 0.001 | Not Included |  |
| Urban area | 0.84 [0.70;1.00] | 0.056 | Not Included |  |
| Nursing homes | 3.31 [2.74;3.98] | <0.001 | NS |  |
| Basal vital signs |  |  |  |  |
| RR, number of breaths/min | 1.05 [1.04;1.06] | <0.001 | NS |  |
| SpO2, % | 0.94 [0.93;0.94] | <0.001 | NS |  |
| FiO2, % | 16.6 [10.2;27.0] | <0.001 | NS |  |
| SaFi | 0.99 [0.99;0.99] | <0.001 | NS |  |
| SBP, mmHg | 0.99 [0.99;0.99] | <0.001 | NS |  |
| DBP, mmHg | 0.98 [0.98;0.99] | <0.001 | NS |  |
| MBP, mmHg | 0.99 [0.98;0.99] | <0.001 | Not Included |  |
| HR, number of beats/min | 1.01 [1.01;1.01] | <0.001 | NS |  |
| Temperature, ºC | 1.12 [1.02;1.22] | 0.017 | Not Included |  |
| GCS, points | 0.84 [0.82;0.85] | <0.001 | NS |  |
| Glucose, mg/dL | 1.00 [1.00;1.00] | <0.001 | NS |  |
| Creatinine, mg/dL | 1.46 [1.40;1.51] | <0.001 | NS |  |
| Lactate, mmol/L | 1.15 [1.13;1.17] | <0.001 | NS |  |
| Outcomes |  |  |  |  |
| mSOFA, points | 1.40 [1.37;1.43] | <0.001 | 1.28 [1.18-1.38] | <0.001 |
| NIRS | 4.85 [3.70;6.35] | <0.001 | 2.06 [1.46-2.91] | <0.001 |
| IRS | 5.79 [4.73;7.09] | <0.001 | 2.93 [2.03-4.23] | <0.001 |
| Noradrenaline use | 9.46 [7.25;12.3] | <0.001 | 1.70 [1.21-2.39] | 0.002 |
| Diagnosis group |  |  |  |  |
| Cardiovascular | Ref. | Ref. |  |  |
| Neurology | 1.33 [1.06;1.68] | 0.014 | NS |  |
| Respiratory | 3.13 [2.45;4.01] | <0.001 | 2.06 [1.52-2.78] | <0.001 |
| Digestive | 1.79 [1.26;2.55] | 0.001 | 1.87 [1.28-2.73] | 0.001 |
| Infection | 3.04 [2.33;3.96] | <0.001 | 1.96 [1.43-2.68] | <0.001 |
| Trauma and injury | 0.87 [0.67;1.14] | 0.313 | 1.65 [1.22-2.22] | 0.001 |
| Poisoning | 0.30 [0.17;0.52] | <0.001 | NS |  |
| Others | 1.83 [1.19;2.82] | 0.006 | NS |  |
| aCCI (points) |  |  | NS |  |
| 0 | Ref. | Ref. |  |  |
| 1 | 2.20 [1.46;3.31] | <0.001 |  |  |
| 2 | 3.66 [2.49;5.37] | <0.001 |  |  |
| 3 | 4.42 [3.09;6.31] | <0.001 |  |  |
| 4 | 8.49 [6.09;11.8] | <0.001 |  |  |
| AIDS | 1.03 [0.51;2.06] | 0.940 | Not Included |  |
| Solid tumor metastatic | 4.41 [3.46;5.63] | <0.001 | 4.28 [3.20-5.73] | <0.001 |
| Liver disease severe | 2.01 [1.46;2.77] | <0.001 | NS |  |
| Lymphoma | 2.00 [1.16;3.47] | 0.013 | Not Included |  |
| Leukemia | 3.34 [2.03;5.49] | <0.001 | 4.21 [2.49-7.11] | <0.001 |
| Solid tumor localized | 2.01 [1.68;2.40] | <0.001 | NS |  |
| DM end organ damage | 1.96 [1.59;2.41] | <0.001 | NS |  |
| Severe CKD | 2.50 [2.05;3.05] | <0.001 | NS |  |
| Hemiplegia | 2.46 [1.86;3.25] | <0.001 | NS |  |
| DM uncomplicated | 1.36 [1.10;1.69] | 0.005 | NS |  |
| Liver disease mild | 1.00 [0.65;1.55] | 0.990 | Not Included |  |
| Peptic ulcer disease | 1.35 [1.05;1.73] | 0.018 | Not Included |  |
| Connective disease | 1.67 [1.28;2.19] | <0.001 | NS |  |
| COPD | 1.64 [1.38;1.95] | <0.001 | NS |  |
| Dementia | 2.83 [2.32;3.44] | <0.001 | NS |  |
| Cerebrovascular disease | 1.83 [1.47;2.29] | <0.001 | NS |  |
| Peripheral vascular disease | 1.51 [1.21;1.89] | <0.001 | NS |  |
| Congestive heart failure | 2.85 [2.40;3.39] | <0.001 | 1.52 [1.25-1.85] | <0.001 |
| Myocardial infarction | 1.49 [1.24;1.79] | <0.001 | NS |  |
| Hospital-inpatient | 5.16 [4.17;6.39] | <0.001 | 2.43 [1.90-3.10] | <0.001 |
| Hospitalization-day | 1.01 [1.01;1.02] | <0.001 | 0.97 [0.96-0.98] | <0.001 |
| ICU-admission | 3.00 [2.48;3.63] | <0.001 | NS |  |
| COVID-19 | 1.93 [1.62;2.31] | <0.001 | 1.33 [1.10-1.61] | <0.001 |

*Abbreviations*: 95%CI: 95% confidence interval; Ref: Reference category for Hazard ratio calculation; Not Included: Not Included in the multivariate model; NS: Not significant; ALS: advanced life support requirement; RR: respiratory rate; SPO2: oxygen saturation; FiO2: fraction of inspired oxygen; SaFi: pulse oximetry saturation/fraction of inspired oxygen ratio; SBP: systolic blood pressure; DBP: diastolic blood pressure; MBP: mean blood pressure (MBP=[(2 x DBP)+ SBP] / 3); HR: heart rate; GCS: Glasgow coma scale; mSOFA: modified Sequential Organ Failure Assessment; NIRS: non-invasive respiratory support; IRS: invasive respiratory support; aCCI: age-adjusted Charlson comorbidity index; AIDS: acquired immunodeficiency syndrome; DM: Diabetes mellitus; CKD: chronic kidney disease; COPD: chronic obstructive pulmonary disease; Hospital-inpatient (admission to hospital); Hospitalization-day (days of hospitalization); ICU: intensive care unit; COVID-19: Coronavirus disease.

^a^The age groups selection was based on both epidemiological and statistical criteria, i.e., our distribution of patients across groups

Table S2. Factors associated to mortality (univariate and multivariate by Cox regression) for those patients with COVID-19

|  | Univariate (log-rank) | | Multivariate (Cox regression) | |
| --- | --- | --- | --- | --- |
| Variable | Hazard ratio (95%CI) | *p* value | Hazard ratio (95%CI) | *p* value |
| Age, year | 1.04 [1.03;1.06] | <0.001 | 1.06 [1.02-1.11] | 0.001 |
| Age groups, year^a^ |  |  | Not Included |  |
| 18-49 | Ref. | Ref. |  |  |
| 50-74 | 1.79 [0.92;3.51] | 0.088 |  |  |
| >75 | 4.94 [2.66;9.17] | <0.001 |  |  |
| Sex, Female | 0.89 [0.65;1.21] | 0.462 | Not Included |  |
| ALS | 0.59 [0.42;0.81] | 0.001 | Not Included |  |
| Urban area | 1.09 [0.76;1.56] | 0.650 | Not Included |  |
| Nursing homes | 3.07 [2.23;4.24] | <0.001 | NS |  |
| Basal vital signs |  |  |  |  |
| RR, number of breaths/min | 1.05 [1.03;1.06] | <0.001 | NS |  |
| SpO2, % | 0.95 [0.94;0.96] | <0.001 | NS |  |
| FiO2, % | 10.5 [3.52;31.5] | <0.001 | 0.01 [0.00-0.50] | 0.020 |
| SaFi | 0.99 [0.99;0.99] | <0.001 | NS |  |
| SBP, mmHg | 0.99 [0.98;0.99] | <0.001 | NS |  |
| DBP, mmHg | 0.98 [0.98;0.99] | <0.001 | 1.01 [1.00-1.02] | 0.025 |
| MBP, mmHg | 0.98 [0.98;0.99] | <0.001 | Not Included |  |
| HR, number of beats/min | 1.01 [1.01;1.02] | <0.001 | NS |  |
| Temperature, ºC | 1.10 [0.95;1.28] | 0.181 | Not Included |  |
| GCS, points | 0.84 [0.81;0.87] | <0.001 | NS |  |
| Glucose, mg/dL | 1.01 [1.00;1.01] | <0.001 | NS |  |
| Creatinine, mg/dL | 1.78 [1.61;1.97] | <0.001 | NS |  |
| Lactate, mmol/L | 1.14 [1.11;1.18] | <0.001 | NS |  |
| Outcomes |  |  |  |  |
| mSOFA, points | 1.39 [1.33;1.45] | <0.001 | 1.40 [1.17-1.68] | <0.001 |
| NIRS | 3.60 [2.20;5.88] | <0.001 | NS |  |
| IRS | 5.47 [3.56;8.41] | <0.001 | NS |  |
| Noradrenaline use | 10.5 [6.50;17.0] | <0.001 | 2.88 [1.37-6.06] | 0.005 |
| Diagnosis group |  |  |  |  |
| Cardiovascular | Ref. | Ref. |  |  |
| Neurology | 0.79 [0.48;1.32] | 0.370 | NS |  |
| Respiratory | 1.77 [1.12;2.80] | 0.014 | 2.41 [1.30-4.45] | 0.004 |
| Digestive | 0.95 [0.47;1.92] | 0.885 | NS |  |
| Infection | 1.95 [1.21;3.14] | 0.006 | NS |  |
| Trauma and injury | 0.83 [0.49;1.40] | 0.480 | NS |  |
| Poisoning | 0.21 [0.05;0.88] | 0.032 | NS |  |
| Others | 1.34 [0.58;3.10] | 0.497 | NS |  |
| aCCI (points) |  |  | NS |  |
| 0 | Ref. | Ref. |  |  |
| 1 | 0.80 [0.30;2.16] | 0.659 | 0.25 [0.07-0.84] | 0.025 |
| 2 | 2.63 [1.24;5.56] | 0.012 | 0.28 [0.08-0.98] | 0.048 |
| 3 | 2.85 [1.45;5.59] | 0.002 | 0.20 [0.04-0.88] | 0.033 |
| 4 | 5.38 [2.87;10.1] | <0.001 | NS |  |
| AIDS | 0.78 [0.25;2.46] | 0.677 | Not Included |  |
| Solid tumor metastatic | 2.90 [1.76;4.80] | <0.001 | 3.73 [1.95-7.12] | <0.001 |
| Liver disease severe | 2.29 [1.36;3.83] | 0.002 | Not Included |  |
| Lymphoma | 4.40 [1.63;11.9] | 0.004 | Not Included |  |
| Leukemia | 7.59 [3.53;16.3] | <0.001 | 10.0 [3.86-26.0] | <0.001 |
| Solid tumor localized | 1.70 [1.20;2.41] | 0.003 | Not Included |  |
| DM end organ damage | 2.37 [1.62;3.48] | <0.001 | NS |  |
| Severe CKD | 2.47 [1.72;3.54] | <0.001 | NS |  |
| Hemiplegia | 3.00 [1.93;4.67] | <0.001 | 1.75 [1.00-3.04] | 0.046 |
| DM uncomplicated | 1.06 [0.69;1.63] | 0.784 | Not Included |  |
| Liver disease mild | 0.92 [0.43;1.97] | 0.833 | Not Included |  |
| Peptic ulcer disease | 1.39 [0.90;2.14] | 0.137 | Not Included |  |
| Connective disease | 1.33 [0.83;2.12] | 0.235 | Not Included |  |
| COPD | 1.41 [1.01;1.98] | 0.045 | Not Included |  |
| Dementia | 2.62 [1.87;3.66] | <0.001 | NS |  |
| Cerebrovascular disease | 1.30 [0.82;2.06] | 0.262 | Not Included |  |
| Peripheral vascular disease | 1.59 [1.05;2.40] | 0.029 | Not Included |  |
| Congestive heart failure | 2.45 [1.79;3.35] | <0.001 | 2.02 [1.33-3.05] | <0.001 |
| Myocardial infarction | 1.27 [0.89;1.79] | 0.185 | Not Included |  |
| Hospital-inpatient | 4.08 [2.71;6.13] | <0.001 | NS |  |
| Hospitalization-day | 1.01 [1.00;1.02] | 0.186 | Not Included |  |
| ICU-admission | 1.94 [1.29;2.94] | 0.002 | Not Included |  |

*Abbreviations*: 95%CI: 95% confidence interval; Ref: Reference category for Hazard ratio calculation; Not Included: Not Included in the multivariate model; NS: Not significant; ALS: advanced life support requirement; RR: respiratory rate; SPO2: oxygen saturation; FiO2: fraction of inspired oxygen; SaFi: pulse oximetry saturation/fraction of inspired oxygen ratio; SBP: systolic blood pressure; DBP: diastolic blood pressure; MBP: mean blood pressure (MBP=[(2 x DBP)+ SBP] / 3); HR: heart rate; GCS: Glasgow coma scale; mSOFA: modified Sequential Organ Failure Assessment; NIRS: non-invasive respiratory support; IRS: invasive respiratory support; aCCI: age-adjusted Charlson comorbidity index; AIDS: acquired immunodeficiency syndrome; DM: Diabetes mellitus; CKD: chronic kidney disease; COPD: chronic obstructive pulmonary disease; Hospital-inpatient (admission to hospital); Hospitalization-day (days of hospitalization); ICU: intensive care unit.

^a^The age groups selection was based on both epidemiological and statistical criteria, i.e., our distribution of patients across groups
